# Decoding Spontaneous Emotional States in the Human Brain

**DOI:** 10.1371/journal.pbio.2000106

**Published:** 2016-09-14

**Authors:** Philip A. Kragel, Annchen R. Knodt, Ahmad R. Hariri, Kevin S. LaBar

**Affiliations:** Department of Psychology & Neuroscience, Duke University, Durham, North Carolina, United States of America; Oxford University, United Kingdom

## Abstract

Pattern classification of human brain activity provides unique insight into the neural underpinnings of diverse mental states. These multivariate tools have recently been used within the field of affective neuroscience to classify distributed patterns of brain activation evoked during emotion induction procedures. Here we assess whether neural models developed to discriminate among distinct emotion categories exhibit predictive validity in the absence of exteroceptive emotional stimulation. In two experiments, we show that spontaneous fluctuations in human resting-state brain activity can be decoded into categories of experience delineating unique emotional states that exhibit spatiotemporal coherence, covary with individual differences in mood and personality traits, and predict on-line, self-reported feelings. These findings validate objective, brain-based models of emotion and show how emotional states dynamically emerge from the activity of separable neural systems.

## Author Summary

Functional brain imaging techniques provide a window into neural activity underpinning diverse cognitive processes, including visual perception, decision-making, and memory, among many others. By treating functional imaging data as a pattern-recognition problem, similar to face- or character-recognition, researchers have successfully identified patterns of brain activity that predict specific mental states; for example, the kind of an object being viewed. Moreover, these methods are capable of predicting mental states in the absence of external stimulation. For example, pattern-classifiers trained on brain responses to visual stimuli can successfully predict the contents of imagery during sleep. This research shows that internally mediated brain activity can be used to infer subjective mental states; however, it is not known whether more complex emotional mental states can be decoded from neuroimaging data in the absence of experimental manipulations. Here we show that brain-based models of specific emotions can detect individual differences in mood and emotional traits and are consistent with self-reports of emotional experience during intermittent periods of wakeful rest. These findings show that the brain dynamically fluctuates among multiple distinct emotional states at rest. More practically, the results suggest that brain-based models of emotion may help assess emotional status in clinical settings, particularly in individuals incapable of providing self-report of their own emotional experience.

## Introduction

Functional neuroimaging offers unique insight into how mental representations are encoded in brain activity [1,2]. Seminal cognitive neuroscience studies demonstrated that distributed patterns of cortical activity measured with functional magnetic resonance imaging (fMRI) contain information capable of differentiating among visual percepts, including object categories [3] and basic visual features [4]. Extending findings from these studies, subsequent work demonstrated that machine learning models trained on stimulus-evoked brain activity, termed “decoding” or “mind-reading” [5], can be used to predict the contents of working memory [6–8] and mental imagery [9,10], even during sleep [11]. Thus, pattern recognition approaches can identify defining features of mental processes, even when driven solely on the basis of endogenous brain activity. The approach was further shown to accurately discriminate among multiple cognitive processes (e.g., decision-making, working memory, response inhibition, among others) in independent subjects [12], establishing the efficacy of assessing diverse mental states with fMRI across individuals.

Paralleling cognitive studies decoding task-evoked brain activity, multivariate decoding approaches have recently been used to map patterns of neural activity evoked by emotion elicitors onto discrete feeling states [13,14]. However, a key piece of missing evidence is whether categorically distinct emotional brain states occur intrinsically [15,16] in the absence of external eliciting stimuli. If so, then it should be possible to classify the emotional status of a human being based on analysis of spontaneous fluctuations of brain activity during rest. Successful classification would validate multivariate decoding of unconstrained brain activity and provides insight into the nature of emotional brain activity during the resting state.

**Fig 1:**
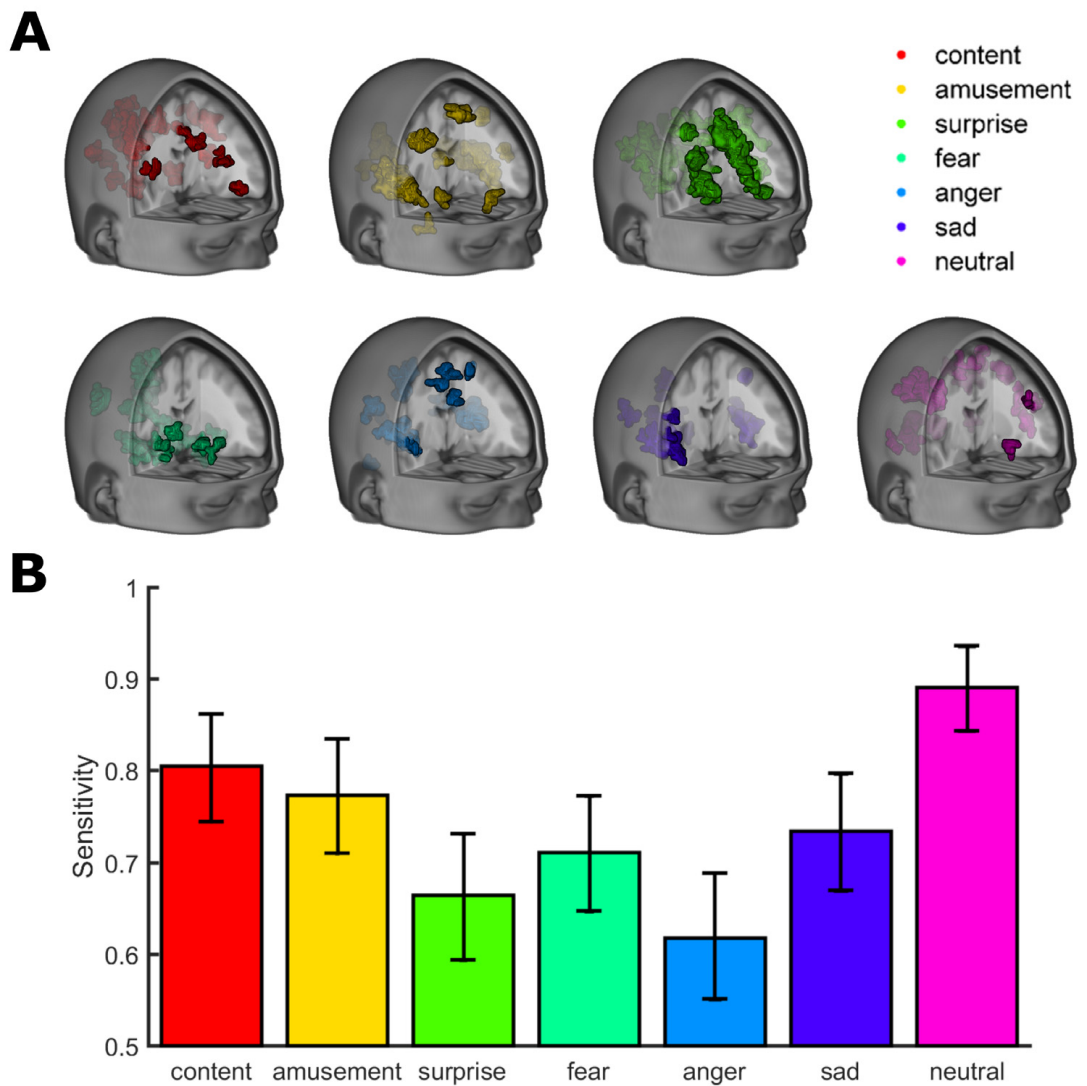
Distributed patterns of brain activity predict the experience of discrete emotions. (A) Parametric maps indicate brain regions in which increased fMRI signal informs the classification of emotional states. See [13] for details of the development and validation of these neural decoding models. (B) Sensitivity of the seven models. Error bars depict 95% confidence intervals. The data underlying this figure can be found in S1 Data.

Adapting the logic of other cognitive imaging studies [16,17], we postulate that the presence of spontaneous emotional brain states should be detectable using multivariate models derived from prior investigations of emotion elicitation. We previously developed decoding algorithms to classify stimulus-evoked responses to emotionally evocative cinematic films and instrumental music [13]. These neural models (Fig 1) accurately classify patterns of neural activation associated with six different emotions (contentment, amusement, surprise, fear, anger, and sadness) and a neutral control state in independent subjects, generalizing across induction modality. Importantly, these neural biomarkers track the subjective experience of discrete emotions independent of differences in the more general dimensions of valence and arousal [18]. By indexing the extent to which a pattern of neural activation to extrinsic stimuli reflects a specific emotion, these models can be used to test whether intrinsic spatiotemporal patterns of brain activity correspond to stimulus-evoked emotional states.

Here, we evaluate whether these neural models of discrete emotions generalize to spontaneous brain activation measured via fMRI in two experiments. The first experiment assesses if model predictions are convergent with individual differences in self-reported mood and emotional traits. Because individual differences are linked to mental health and subjective well-being [19–21], this evaluation provides insight into the potential clinical utility of quantifying spontaneous emotional states, as they may be associated with risk factors for mental illness. The second experiment employs an experience sampling procedure to evaluate whether model predictions based on brain activity during periods of rest are congruent with on-line measures of emotional experience. Together, these studies probe how brain-based models of specific emotion categories quantify changes in extemporaneous affect both between and within individuals.

## Results

### Classification of Resting-State Brain Activity

**Fig 2:**
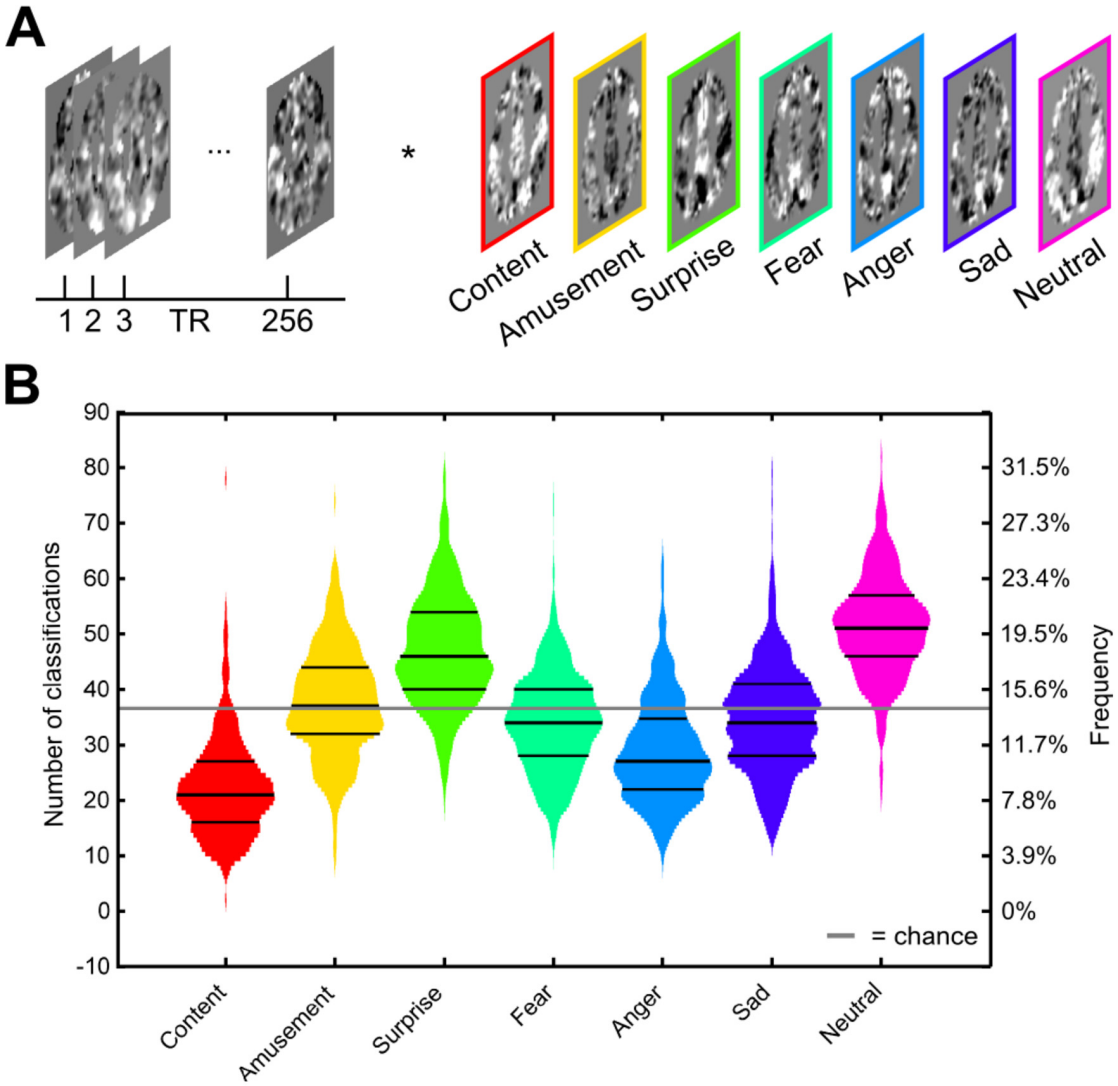
Emotional states emerge spontaneously during resting-state scans. (A) Procedure for classification of resting-state data. Scores are computed by taking the scalar product of preprocessed data and regression weights from decoding models. (B) Frequency distributions for the classification of all seven emotional states (*n* = 499). The mean, 25th, and 75th percentiles are indicated by black lines. The solid gray line indicates the number of trials that would occur from random guessing. The data underlying this figure can be found in S1 Data. The raw fMRI resting state data can be obtained from https://www.haririlab.com/projects.

We applied the multivariate models of emotional experience to brain activation acquired from young adults during resting-state fMRI (*n* = 499; Fig 2A). Two consecutive runs of resting-state scans were acquired, spanning a total duration of 8.53 min. Following preprocessing of data, we computed the scalar product of the resting-state signal and emotion category-specific model weights at every time point of data acquisition. This procedure yielded scores that reflect the relative evidence for each of seven emotional states across the full scanning period. A confirmatory analysis revealed that voxels distributed across the whole brain informed this prediction, as opposed to activity in a small number of brain regions (S1 Fig).

If emotional brain states occur spontaneously, the frequency of classifications from our decoding models should be more varied than the uniform distribution that would be expected by chance. To test this hypothesis, we sought to identify whether the total time (or absolute frequency) in each state differed across emotion categories. Such an analysis informs the degree to which discrete emotional brain states may spontaneously occur and, by extension, could contribute to the identification of individual differences that map onto the likelihood of experiencing specific spontaneous states. To perform this comparison, we identified the single model with the maximum score at each time point (one-versus-all classification) and summed the number of time points assigned to each category. The frequency of emotional states clearly differed across categories (Fig 2B, χ^2^ = 1491.52, *P* < .0001, Friedman test), in contrast to the uniform distribution that would be expected if emotional brain-states did not occur in spontaneous activity (see S2 Fig).

Follow-up comparisons revealed that neutral states occurred more frequently than chance rates (20.1 ± 3.59% [s.d.], *z* = 20.50, *P*_unc_ = 2.03E-93), followed by states of surprise (18.37 ± 3.87% [s.d.], *z* = 16.38, *P*_unc_ = 2.47E-60) and amusement (14.71 ± 3.78% [s.d.], *z* = 1.25, *P*_unc_ = 0.21). States of sadness (13.49 ± 3.76% [s.d.], *z* = -3.31, *P*_unc_ = 9.24E-4), fear (13.26 ± 3.42% [s.d.], *z* = -5.28, *P*_unc_ = 1.28E-7), and anger (11.31 ± 3.62% [s.d.], *z* = -13.07, *P*_unc_ = 4.78E-39) occurred with lower frequency, while states of contentment occurred the least often (8.74% ± 3.42% [s.d.], *z* = -19.61, *P*_unc_ = 1.33E-85; see Table 1).

**Table 1:** Pairwise comparisons of classification frequency ranks for the emotion models.

Model 1	Model 2	Lower Bound	Estimate	Upper Bound	*P*_unc_
Content	Amusement	-2.836	-2.422	-2.008	2.601E-69
Content	Surprise	-4.199	-3.785	-3.370	2.310E-168
Content	Fear	-2.199	-1.785	-1.370	7.583E-38
Content	Anger	-1.445	-1.031	-0.617	8.165E-13
Content	Sad	-2.291	-1.877	-1.463	8.186E-42
Content	Neutral	-4.771	-4.357	-3.943	7.192E-223
Amusement	Surprise	-1.777	-1.363	-0.949	3.265E-22
Amusement	Fear	0.223	0.637	1.051	6.157E-05
Amusement	Anger	0.977	1.391	1.805	4.001E-23
Amusement	Sad	0.131	0.545	0.959	1.335E-03
Amusement	Neutral	-2.349	-1.935	-1.521	2.045E-44
Surprise	Fear	1.586	2.000	2.414	1.999E-47
Surprise	Anger	2.339	2.754	3.168	1.979E-89
Surprise	Sad	1.494	1.908	2.322	3.404E-43
Surprise	Neutral	-0.986	-0.572	-0.158	5.662E-04
Fear	Anger	0.339	0.754	1.168	6.790E-07
Fear	Sad	-0.506	-0.092	0.322	1.000E+00*
Fear	Neutral	-2.986	-2.572	-2.158	4.128E-78
Anger	Sad	-1.260	-0.846	-0.432	1.151E-08
Anger	Neutral	-3.740	-3.326	-2.912	3.629E-130
Sad	Neutral	-2.894	-2.480	-2.066	1.189E-72

*P*_unc_ = uncorrected *P* value.

*does not survive FDR correction for multiple comparisons. Estimates reflect the difference in rank for Model 1 versus Model 2

Although patterns of neural activation were most often classified as neutral as a whole, it is possible that consistent fluctuations in the time course of emotional states occur against this background. Research on MRI scanner-related anxiety has shown that self-report [22,23] and peripheral physiological [24] measures of anxiety peak at the beginning of scanning, when subjects first enter the scanner bore. This literature predicts that brain states indicative of fear should be most prevalent at the beginning of resting-state runs, and that neutral states should emerge over time, given their overall high prevalence (Fig 2B).

**Fig 3:**
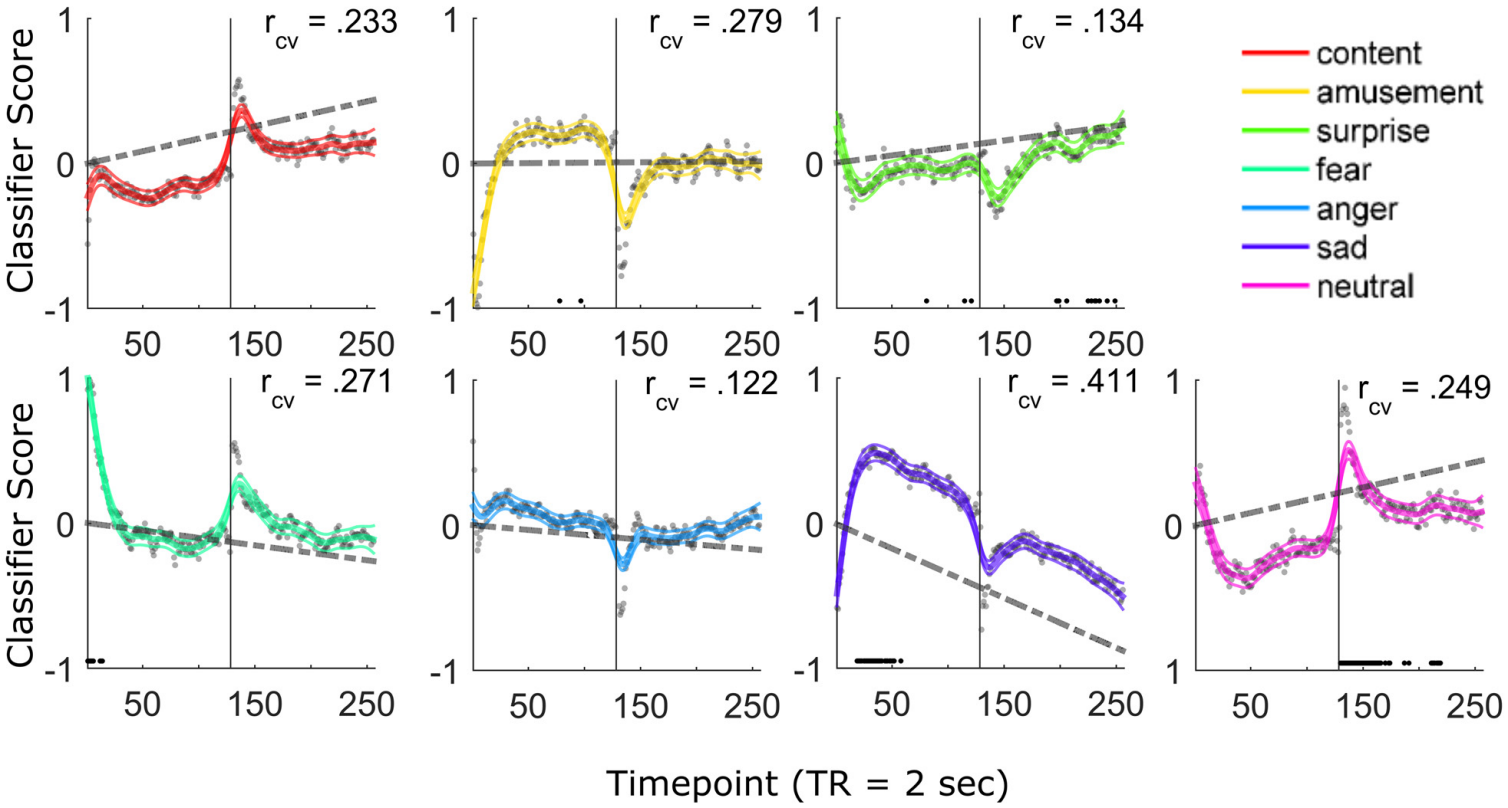
Emotional states exhibit coherence during resting-state scans. Gray circles reflect the sample mean classification scores for all seven emotions (*n* = 499). Thick colored lines display group-average predicted time course using smoothing splines (with bordering 95% confidence interval). Text overlay (r_cv_) indicates the average cross-validated correlation between splines fitted for each subject and tested on the average fit of other subjects. Dashed lines indicate linear fits over time. Solid black dots indicate time points at which a model has the highest proportion of classifications. Data are concatenated across two sessions of 256 s (solid vertical line). Note the early peak for fear scores and general increases in neutral scores over time. The data underlying this figure can be found in S1 Data. The raw fMRI resting state data can be obtained from https://www.haririlab.com/projects.

To assess gradual changes in the emotional states over time, we performed Friedman tests separately for each emotion category, all of which revealed significant effects of time (see S1 Table). Next, we quantified the direction of these effects using general linear models to predict classifier scores using scan time as an input. We found the scores for fear decreased over time ($\hat{\beta }=-0.001$, t_498_ = -4.92, *P*_unc_ = 1.20E-006, Fig 3 gray lines), whereas neutral states exhibited an increasing trend throughout the scanning period ($\hat{\beta }=0.0017$, t_498_ = 7.36, *P*_unc_ = 7.66E-013), consistent with predictions (additional effects were observed for scores for contentment [$\hat{\beta }=0.0017$, t_498_ = 7.37, *P*_unc_ = 7.05E-13], surprise [$\hat{\beta }=0.0010$, t_498_ = 4.07, *P*_unc_ = 5.51E-05], anger [$\hat{\beta }=-0.0007$, t_498_ = -3.36, *P*_unc_ = 0.00085], and sadness [$\hat{\beta }=-0.0034$, t_498_ = -15.59, *P*_unc_ < 2.52E-038]).

To determine whether emotional states exhibited consistent dynamics over the course of the scanning period, we fit smoothing spline models [25] for each subject and assessed the correlation between each subject and the average time course of other subjects in a cross-validation procedure. This analysis showed that there is substantial moment-to-moment variability in the time course of emotional states across subjects (which cannot simply be explained by scaling differences in the emotion models or resting-state data; see S3 Fig). Consistent with the linear models using time as a predictor, evidence for neutral brain states was most prevalent in the second scanning session, especially during a peak at the beginning of the run, whereas the time course for fear peaked at the beginning of the first run and decreased throughout the scanning session. The model for surprise exhibited a similar time course as neutral states but peaked at the end of the second run. Additionally, this analysis showed that evidence for sad classifications peaked in the middle of the first run and decreased over time. Overall, these time series revealed a gradual change in evidence from negative emotions (fear and sadness in run 1) to non-valenced or bi-valenced emotions (neutral and surprise in run 2).

To ensure that our emotion-specific brain states are not proxies for more general resting-state networks thought to subserve other functions, we examined the spatial overlap between our models and those commonly derived by connectivity-based analysis of resting-state fMRI data [26]. On average, we observed little overlap (Jaccard index = 13.1 ± 1.97% [s.d.]; range 10.8%–16.7%) with the seven most prominent networks found in resting-state data, implicating a substantial degree of independence.

**Fig 4:**
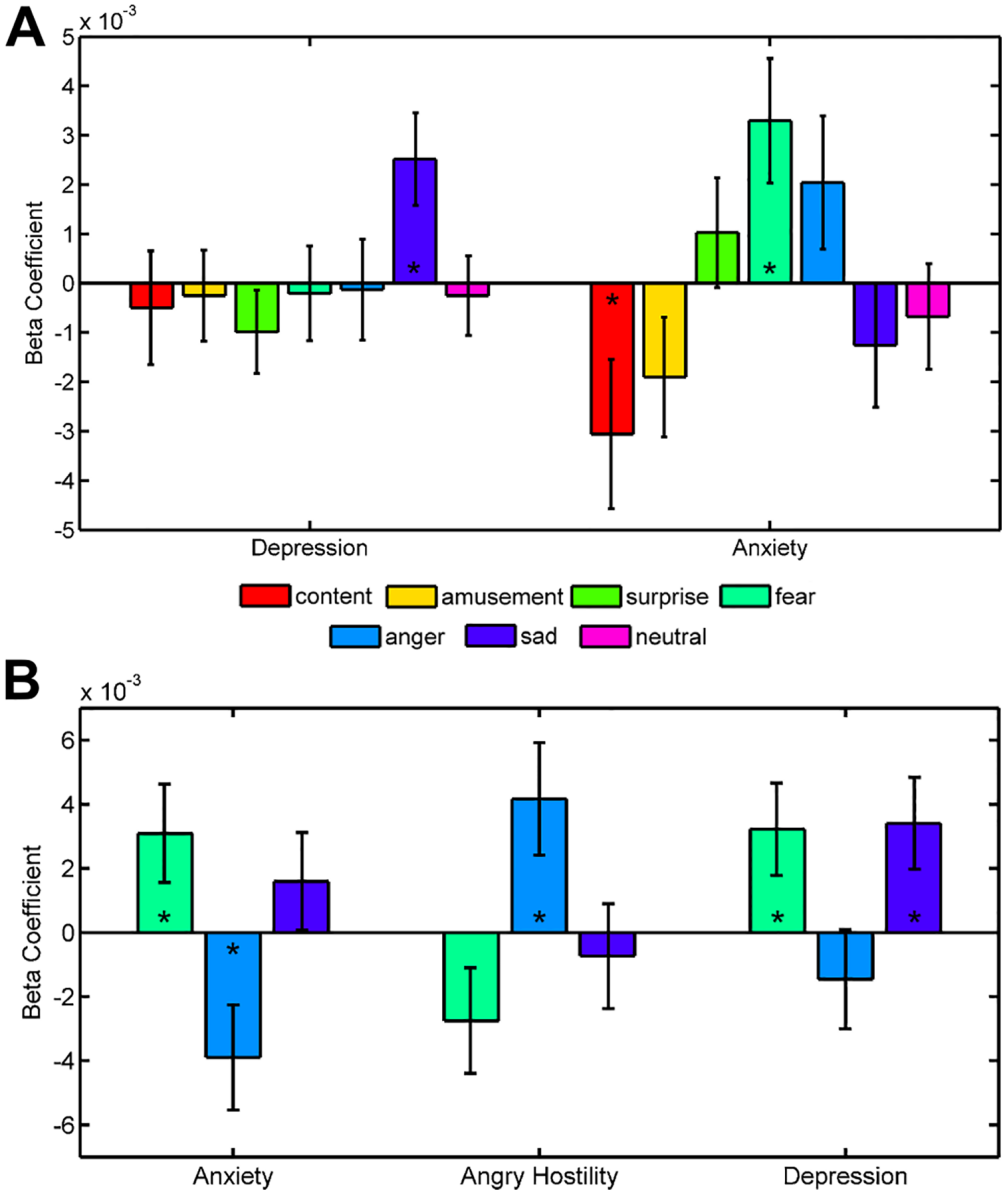
Individual differences in mood and personality modulate the occurrence of spontaneous emotional brain states. (A) Differences in depressive and anxious mood are associated with increases in the frequency of sad and fear classifications during rest. (B) Emotional traits of Anxiety, Angry Hostility, and Depression track differences in the frequency of fear, anger, and sad classifications (*n* = 499, error bars reflect standard error, * indicates effects significant at *P*_unc_ < .05). The data underlying this figure can be found in S1 Data. The raw fMRI resting state data can be obtained from https://www.haririlab.com/projects.

To further establish the construct validity of the spontaneous emotional brain states, we reasoned that their incidence should vary with individual differences in self-reported mood and personality traits associated with specific emotions. We assayed depressive mood with the Center for Epidemiologic Studies Depression Scale (CESD) [27] and state anxiety using the State-Trait Anxiety Inventory State Version (STAI-S) [28], instructing participants to indicate how they felt during the resting-state scan itself. Binomial regression models revealed that higher depression scores were associated with increases in the frequency of sadness ($\hat{\beta }=0.0025$, t_497_ = 2.673, *P*_unc_ = .0075, Fig 4A, see S4 Fig for scatter plots of predictions) and no other emotional state (all *P*_unc_ > .24). State anxiety was associated with increasing classifications of fear ($\hat{\beta }=0.0033$, t_497_ = 2.608, *P*_unc_ = .0091) and decreasing frequency of contentment ($\hat{\beta }=-0.0031$, t_497_ = -2.015, *P*_unc_ = .0439). Viewing these beta estimates as odds ratios (computed as ${\text{e}}^{\hat{\beta }}$) reveals how a one-unit increase in self-reported mood is associated with differences in the occurrence of spontaneous emotional states. Applying this approach to CESD scores reveals that individuals with a score of 16 (the cutoff for identifying individuals at risk for depression) have 5.92% increased odds of being in a sad state compared to those with a score of 0. In more practical terms, this corresponds to approximately seven extra minutes a day of exhibiting a brain state that would be classified as sadness.

Drawing from the Revised NEO Personality Inventory (NEO-PI-R) [29], we focused personality trait assessment on the specific Neuroticism subfacets of Anxiety, Angry Hostility, and Depression, due to their discriminant validity [30], heritability [31], universality [32], and close theoretical ties to the experience of fear, anger, and sadness. We found that increasing Anxiety scores were associated with more frequent classification of fear ($\hat{\beta }=0.003$, t_497_ = 1.978, *P*_unc_ = 0.0479, Fig 4B) and fewer classifications of anger ($\hat{\beta }=-0.004$, t_497_ = -2.407, *P*_unc_ = 0.0161). Angry Hostility scores were positively associated with the number of anger classifications ($\hat{\beta }=0.0042$, t_497_ = 2.400, *P*_unc_ = 0.0164). Depression scores were positively associated with the frequency of fear ($\hat{\beta }=0.003$, t_497_ = 2.058, *P*_unc_ = 0.0396) and sadness ($\hat{\beta }=0.0037$, t_497_ = 2.546, *P*_unc_ = 0.0109). These results provide converging evidence across both state and trait markers that individual differences uniquely and differentially bias the spontaneous occurrence of brain states indicative of fear, anger, and sadness.

### Concordance with Subjective Experience

**Fig 5:**
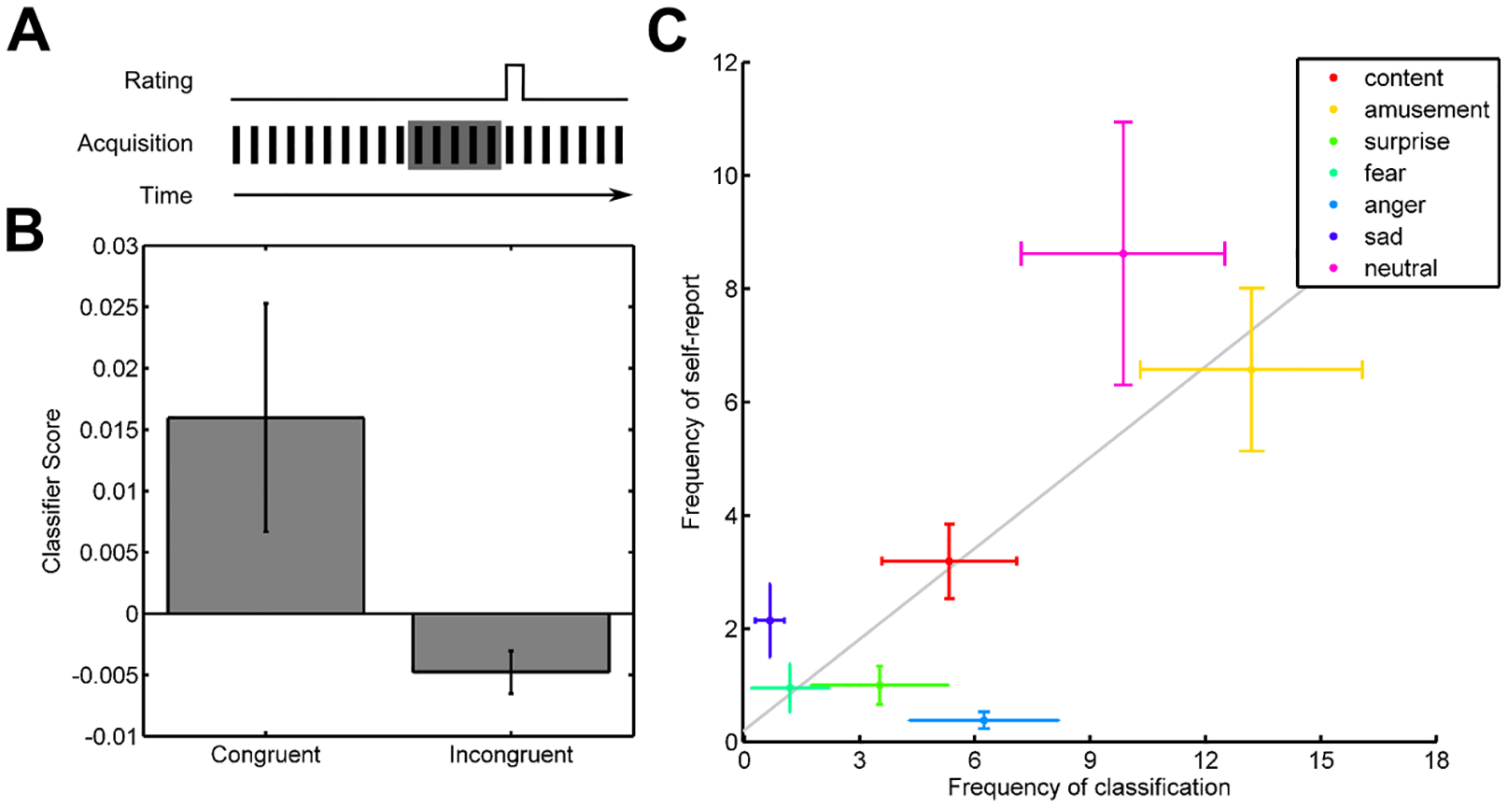
Spontaneous emotional brain states exhibit correspondence with self-report. (A) Participants (*n* = 21) participated in an experience sampling task in which they reported their current emotional state at random intervals exceeding 30 s during fMRI scanning. The five samples of data (lasting 10 s, TR = 2 s) preceding each rating were used to compute predictions of emotional state using multivariate decoding models. (B) Scores for classification models congruent with self-report are greater than incongruent models (*z* = 2.311, *P*_unc_ = 0.0208; Wilcoxon signed rank test). Classification scores are calculated based on the inner product of neural activity and classifier weights and indicate the relative evidence for the different emotion models. (C) The frequency of classifications from multivariate models significantly correlates with those made by participant self-report (r = .3876 ± 0.102 [s.e.m.], t_20_ = 2.537, *P*_unc_ = .0196; one sample *t* test). Gray line indicates best-fitting least-squares line for group mean. In all panels, error bars reflect standard error of the mean. The data underlying this figure can be found in S1 Data.

Finally, we examined whether the predictions of our decoding models were consistent with self-report of emotional experience during periods of unconstrained rest. We conducted a separate fMRI experiment in which an independent sample of young adult participants (*n* = 21) performed an experience sampling task in the absence of external stimulation (Fig 5A). Participants were instructed to rest and let their mind wander freely with their eyes open during scanning. Following intervals of rest of at least 30 s, a rating screen appeared during which participants moved a cursor to the location on the screen that best indicated how they currently felt.

If spontaneous emotional states are accessible to conscious awareness, then scores should be greater for emotion models congruent with self-report relative to scores for models incongruent with self-report. Contrasting emotion models in this manner is advantageous from a signal detection standpoint because it minimizes noise by averaging across emotions, as some were reported infrequently or not at all in some subjects (see [33] for an analogous approach to predict the contents of memory retrieval during similarly unconstrained free-recall). To test our hypothesis, we extracted resting-state fMRI data from the 10-s interval preceding each self-report query and applied multivariate models to determine the extent to which evidence for the emotional brain states in this window predicted the participants’ conscious emotional experience.

Consistent with our hypothesis, we found that scores for models congruent with self-report were positive (0.016 ± 0.0093 [s.e.m.], *z* = 2.068, *P*_unc_ = 0.0386; Wilcoxon signed rank test), whereas scores for incongruent models were negative (-0.0048 ± 0.0017 [s.e.m.], *z* = -3.041, *P*_unc_ = 0.0024). Classification of individual trials into the seven emotion categories exhibited an overall accuracy of 27.9 ± 2.1% (s.e.m.) of trials, where chance agreement is 21.47% (*P*_unc_ = 0.001; binomial test). Not only do these results demonstrate that classification models are sensitive to changes in emotional state reported by participants, but also that there is selectivity in their predictions, as negative scores indicate evidence against emotion labels that are incongruent with self-report. Establishing both sensitivity and selectivity is important for the potential use of these brain-based models as diagnostic biomarkers of emotional states.

As an additional validation of our decoding models, we examined the correspondence between the prevalence of individual emotional brain states as detected via pattern classification and participant self-report. Classifications based on self-report and multivariate decoding yielded similar frequency distributions (Fig 5C), in which neutral and amusement were the most frequent. We found a positive correlation between the frequency of classifications based on participant ratings and multivariate decoding (r = .3876 ± 0.102 [s.e.m.], t_20_ = 2.537, *P*_unc_ = .0196; one sample *t* test), further demonstrating a link between patterning of brain states and subjective ratings of emotional experience in the absence of external stimuli or contextual cues.

## Discussion

Converging findings from our experiments provide evidence that brain states associated with distinct emotional experiences emerge during unconstrained rest. Whereas prior work has decoded stimulus-evoked responses to emotional events, our study demonstrates that spontaneous neural activity dynamically fluctuates among multiple emotional states in a reliable manner over time. Observing such coherent, emotion-specific patterns in spontaneous fMRI activation provides evidence to support theories that posit emotions are represented categorically in the coordinated activity of separable neural substrates [34,35].

Validating the neural biomarkers in the absence of external stimulation suggests that they track information of functional significance, and do not merely reflect properties of the stimuli used in their development. It is possible that these classifiers detect the endogenous activity of distributed neural circuits, consistent with recent views that emotions are not represented in modular functional units [36,37]. However, the extent to which such activity is the result of innate emotion-dedicated circuitry, a series of cognitive appraisals, or constructive processes shaped by social and environmental factors remains to be determined (for a review of these viewpoints, see [38]). Regardless of the relative influence of such factors, the present findings suggest that the emotion-specific biomarkers track the expression of functionally distinct brain systems, as opposed to idiosyncrasies of the particular machine-learning problem.

Our findings complement recent studies demonstrating that a variety of emotion manipulations have lasting effects on resting brain activity [39–41]. For instance, one study revealed elevated striatal activity following gratifying outcomes in a decision-making task—an effect that was diminished in individuals with higher depressive tendencies [39]. Because these effects immediately followed emotional stimulation, they could plausibly reflect regulatory processes or lingering effects of mood. The present results, on the other hand, show that resting brain activity transiently fluctuates among multiple emotional states and that these fluctuations vary depending on the emotional status of an individual. Thus, emotional processes unfolding at both long and short time scales likely contribute to spontaneous brain activity.

Findings from our resting state experiment stand in contrast to recent work investigating emotion-specific functional connectivity [42]. In this study, whole-brain resting-state functional connectivity was assessed using seeds identified from a meta-analytic summary of emotion research [43]. This latter approach failed to reveal unique patterns of resting-state connectivity for individual emotions but showed that seed regions were commonly correlated with domain-general resting-state networks, such as the salience network [44]. In light of the present results, it is important to consider methodological differences between studies. Seed-based correlation highlights connectivity between brain regions whose time course of activation is maximally similar to the activity of a small number of voxels (which are averaged together to create a single time series), whereas pattern classification identifies combinations of voxels that maximally discriminate among mental states. Because individual voxels sample diverse neural populations [45], it is plausible that seed-based correlation is biased towards identifying networks that have large amplitudes in seeded regions as opposed to exhibiting specificity (e.g., see [46]). Thus, our approach may have greater sensitivity to detect discriminable categorical patterns.

Results of the experience sampling study provide external validation of our emotion-specific biomarkers [13]. Consistent with the resting-state study, the overall distribution of emotional states was clearly non-uniform, and classifications of neutral states occurred with high frequency. Beyond these commonalities, the inclusion of behavioral self-report led to differences in emotion-related brain activity. States of contentment and amusement were more frequently predicted during experience sampling compared to resting-state (46.31% versus 23.45%), a finding that was corroborated by higher ratings for these emotions in the self-report data. It is possible that this difference in the frequency of positive brain states is the result of a self-presentation bias [47], wherein participants may have employed emotion regulation in order to project a more positive image. Alternatively, it is possible that the self-reporting task requirement elicited more introspection between trials, which contributed to the pattern of altered emotional states [48]. Future work will be necessary to fully characterize how such cognitive-emotional interactions shape the landscape of emotional brain states [36,49].

We found that individual differences in mood states and personality traits are associated with the relative incidence of brain states associated with fear, anger, and sadness. These findings further establish the construct validity of our brain-based models of emotion and link subfacets of Neuroticism to the expression of emotion-specific brain systems. Given their sensitivity to individual differences linked to the symptomology of anxiety and depression, spontaneous emotional brain states may serve as a novel diagnostic tool to determine susceptibility to affective illness or as an outcome measure for clinical interventions aimed at reducing the spontaneous elicitation of specific emotions. This tool may be particularly useful to objectively assess the emotional status of individuals who do not have good insight into their emotions, as in alexithymia, or for those who cannot report on their own feelings, including patients in a vegetative or minimally conscious state.

## Materials and Methods

### Ethics Statement

All participants provided written informed consent in accordance with the National Institutes of Health guidelines as approved by the Duke University IRB. The resting state experiment was approved as part of the Duke Neurogenetics Study (Pro00019095) with an associated database (Pro00014717). The experience sampling project was approved separately (Pro00027404).

### Neural Biomarkers of Emotional States

Classification of emotional states was performed using neural biomarkers that were developed based on blood oxygen level dependent (BOLD) responses to cinematic films and instrumental music [13]. This induction procedure was selected because it reliably elicits emotional responses over a 1 to 2 min period, as opposed to longer-lasting moods. These models were developed to identify neural patterning specific to states of contentment, amusement, surprise, fear, anger, and sadness (in addition to a neutral control state). These particular emotions were modeled to broadly sample both valence and arousal, as selecting common sets of basic emotions (e.g., fear, anger, sadness, disgust, and happiness) undersamples positive emotions. In selecting these particular emotions, we verified that the accuracy of these models tracked the experience of specific emotion categories (average *R*^2^ across emotions = .57) independent of subjective valence and arousal. Thus, the models offer unique insight into the emotional state of individuals and characterize the likelihood they would endorse each of the seven emotion labels, independent of general factors such as valence or arousal.

### Resting-State Experiment

A total of 499 subjects (age = 19.65 ± 1.22 years [mean ± s.d.], 274 women) were included as part of the Duke Neurogenetics Study (DNS), which assesses a wide range of behavioral and biological traits among healthy, young adult university students. For access to this data, see information provided in S1 Text. This sample was independent of that used to develop the classification models. This sample size is sufficient to reliably detect (β = .01) a moderate effect (r = .2) with a type-I error rate of .05, which is particularly important when studying individual differences in neural activity. All participants provided informed consent in accordance with Duke University guidelines and were in good general health. The participants were free of the following study exclusions: (1) medical diagnoses of cancer, stroke, head injury with loss of consciousness, untreated migraine headaches, diabetes requiring insulin treatment, chronic kidney or liver disease, or lifetime history of psychotic symptoms; (2) use of psychotropic, glucocorticoid, or hypolipidemic medication; and (3) conditions affecting cerebral blood flow and metabolism (e.g., hypertension). Diagnosis of any current DSM-IV Axis I disorder or select Axis II disorders (antisocial personality disorder and borderline personality disorder), assessed with the electronic Mini International Neuropsychiatric Interview [50] and Structured Clinical Interview for the DSM-IV subtests [51], were not an exclusion, as the DNS seeks to establish broad variability in multiple behavioral phenotypes related to psychopathology. No participants met criteria for a personality disorder, and 72 (14.4%) participants from our final sample met criteria for at least one Axis I disorder (10 Agoraphobia, 33 Alcohol Abuse, 3 Substance Abuse, 25 Past Major Depressive Episode, 5 Social Phobia). However, as noted above, none of the participants were using psychotropic medication during the course of the DNS.

Participants were scanned on one of two identical 3 Tesla General Electric MR 750 system with 50-mT/m gradients and an eight channel head coil for parallel imaging (General Electric, Waukesha, Wisconsin, USA). High-resolution 3-dimensional structural images were acquired coplanar with the functional scans (repetition time [TR] = 7.7 s; echo time [TE] = 3.0 ms; flip angle [α] = 12°; voxel size = 0.9 × 0.9 × 4 mm; field of view [FOV] = 240 mm; 34 contiguous slices). For the two 4 min, 16 s resting-state scans, a series of interleaved axial functional slices aligned with the anterior commissure—posterior commissure plane were acquired for whole-brain coverage using an inverse-spiral pulse sequence to reduce susceptibility artifact (TR = 2000 ms; TE = 30 ms; α = 60°; FOV = 240 mm; voxel size = 3.75 × 3.75 × 4 mm; 34 contiguous slices). Four initial radiofrequency excitations were performed (and discarded) to achieve steady-state equilibrium. Participants were shown a blank gray screen and instructed to lie still with their eyes open, think about nothing in particular, and remain awake.

Preprocessing of all resting-state fMRI data was conducted using SPM8 (Wellcome Department of Imaging Neuroscience). Images for each subject were slice-time-corrected, realigned to the first volume in the time series to correct for head motion, spatially normalized into a standard stereotactic space (Montreal Neurological Institute template) using a 12-parameter affine model (final resolution of functional images = 2 mm isotropic voxels), and smoothed with a 6 mm FWHM Gaussian filter. Low-frequency noise was attenuated by high-pass filtering with a 0.0078 Hz cutoff.

### Experience Sampling Experiment

A total of 22 subjects (age = 26.04 ± 5.16 years [mean ± s.d.], 11 women) provided informed consent and participated in the study. Data from one participant was excluded from analyses because of excessive head movement (in excess of 1 cm) during scanning. While no statistical test was performed to determine sample size a priori, this sample size is similar to those demonstrating a correspondence between self-report of affect and neural activity [13,52,53].

Participants engaged in an experience sampling task in which they rated their current feelings during unconstrained rest. Participants were instructed to keep their eyes open and let their mind wander freely and that a rating screen [54] would occasionally appear, which they should use to indicate the intensity of the emotion that best describes how they currently feel. This validated assay of emotional self-report consists of 16 emotion words organized radially about the center of the screen. Four circles emanate from the center of the screen to each word (similar to a spoke of a wheel), which were used to indicate the intensity of each emotion by moving the cursor about the screen. During four runs of scanning, participants completed 40 trials (10 per run) with an inter-stimulus interval (ISI) of 30 s plus pseudo-random jitter (Poisson distribution, λ = 4 s).

Self-report data were transformed from two-dimensional cursor locations to categorical labels. Polygonal masks were created by hand corresponding to each emotion term on the response screen. A circular mask in the center of the screen was created for neutral responses. Because terms in the standard response screen did not perfectly match those in the neural models, the item “relief” was scored as “content,” whereas “joy” and “satisfaction” were scored as “amusement.” The items “surprise,” “fear,” “anger,” “sadness,” and “neutral” were scored as normal.

Scanning was performed on a 3 Tesla General Electric MR 750 system with 50-mT/m gradients and an eight channel head coil for parallel imaging (General Electric, Waukesha, Wisconsin, USA). High-resolution images were acquired using a 3D fast SPGR BRAVO pulse sequence (TR = 7.58 ms; TE = 2.936 ms; image matrix = 256^2^; α = 12°; voxel size = 1 × 1 × 1 mm; 206 contiguous slices) for coregistration with the functional data. These structural images were aligned in the near-axial plane defined by the anterior and posterior commissures. Whole-brain functional images were acquired using a spiral-in pulse sequence with sensitivity encoding along the axial plane (TR = 2000 ms; TE = 30 ms; image matrix = 64 × 64; α = 70°; voxel size = 3.8 × 3.8 × 3.8 mm; 34 contiguous slices). Four initial radiofrequency excitations were performed (and discarded) to achieve steady-state equilibrium.

Processing of MR data was performed using SPM8 (Wellcome Department of Imaging Neuroscience). Functional images were slice-time-corrected, spatially realigned to correct for motion artifacts, coregistered to high resolution anatomical scans, and normalized to Montreal Neurologic Institute (MNI) space using high-dimensional warping implemented in the VBM8 toolbox (http://dbm.neuro.uni-jena.de/vbm.html). Low-frequency noise was attenuated by high-pass filtering with a 0.0078 Hz cutoff.

### Statistical Analysis

To rescale data for classification, preprocessed time series were standardized by subtracting their mean and dividing by their standard deviation. Maps of partial least squares (PLS) regression coefficients from stimulus-evoked decoding models [13] were resliced to match the voxel size of functional data. These coefficients are conceptually similar to those in multiple linear regression, only they are computed by identifying a small number of factors (reducing the dimensionality of the problem) that maximize the covariance between patterns of neural activation and emotion labels (for specifics on their computation, see [55]). Classifier scores were computed by taking the scalar product of functional data at each time point and PLS regression coefficients from content, amusement, surprise, fear, anger, sad, and neutral models. Individual time points were assigned categorical labels by identifying the model with the maximal score.

In order to determine if relatively focal or diffuse patterns of resting-state activity informed classification, we computed importance maps for each subject (S1 Fig). This was accomplished by calculating the voxel-wise product between PLS regression coefficients for each emotion model and the average activity of acquisition time points labeled as the corresponding emotion. We made inference on these maps by conducting a mass-univariate one-sample *t* test for each of the seven models, thresholding at FDR *q* = .05.

To address the potential overlap of the emotion classification models and canonical resting-state networks of the brain, we computed the maximal Jaccard index for each emotion model and the seven most prominent resting-state networks identified in Yeo et al [26]. This index is computed as the intersection of voxels in the two maps (voxels above threshold in both maps) relative to their union (the number of voxels above threshold in either map). Thresholds for classification models were adaptively matched to equate the proportion of voxels assigned to each resting state network.

When conducting inferential tests on classification frequency (count data), non-parametric tests were conducted. To test whether classifications were uniformly distributed across the emotion categories, a Friedman test was performed (*n* = 499 subjects, k = 7 emotions). Wilcoxon signed-rank tests were performed to test for differences in frequency relative to chance rates (14.3%) in addition to pairwise comparisons between emotion models, and corrected for multiple comparisons based on the false-discovery rate.

Because the models have different levels of accuracy when used for seven-way classification [13], we additionally conducted wavelet resampling of classifier scores in the time domain [33,56] over 100 iterations to ensure that differences in the sensitivity of models did not bias results. This procedure involved scrambling the wavelet coefficients (identified using the discrete wavelet transform) of classifier scores (time series in Fig 3) to generate random time series with similar autocorrelation as the original data. Classifications were then made on these surrogate time series, and Friedman tests were performed to test for differences in frequencies across categories. This procedure yielded a null distribution for the chi-square statistic against which the observed statistic on unscrambled data was compared.

To test whether classifier scores changed over time, Friedman tests were conducted on the outputs of the emotion models separately (concatenating the time series across runs), as classifier scores were found to violate assumptions of normality. Follow-up tests on the direction of these changes (either as increases or decreases) were conducted using general linear models with one constant regressor and another for linearly increasing time for each subject. Inference on the parameter estimate for changes over time was made using a one-sample *t* test (498 degrees of freedom).

In addition to testing gradual changes over time, smoothing spline models [25] were used to characterize more complex dynamics of emotional states. Because spline models are flexible and may include a different number of parameters for each subject, cross-validation was conducted to assess the coherence of spline fits across subjects. In this procedure, a smoothing spline model was fit for each subject, and its Pearson correlation with the mean fit for all other subjects was computed. The average of resulting correlations accordingly reflects the coherence of nonlinear changes in emotional states across all subjects.

The influence of individual differences in mood and personality was assessed using generalized linear models with a binomial distribution and a logit link function. Multiple models were constructed, each using a single measure from either the CESD, STAI, or facets from the NEO-PI-R to predict the frequency of classifications for the seven emotion categories (seven models per self-report measure). Inference on parameter estimates (characterizing relationships between individual difference measures and classification frequency) was made using a *t* distribution with 497 degrees of freedom.

To control for multiple comparisons, FDR correction (*q* = .05) [57,58] was applied for targeted predictions. For individual differences in mood, this procedure included correction for positive associations between the frequency of sad classifications and CESD scores and between fear classification and STAI values (*P*_thresh_ = .0091). For differences in emotional traits, correction was applied to models predicting the frequency of fear classification on the basis of Anxiety scores, anger classification using Angry Hostility scores, and sad classifications on the basis of Depression scores (*P*_thresh_ = .0479). Scatterplots and predicted outcomes for these regression analyses are displayed in S4 Fig.

To assess concordance in the experience sampling study, classifier scores were averaged for trials congruent and incongruent with self-report for each subject. For instance, all trials in which a participant self-reported “fear,” the classifier outputs from the neural model predicting fear were considered congruent, whereas the remaining six models were averaged as incongruent. Because the frequency of self-report varied across emotions (e.g., endorsement of fear and sadness were very infrequent), scores were averaged across all trials to reduce noise.

In a supplemental analysis, scores were extracted separately for all trials and classified by identifying the model with the highest score. Accuracy was assessed on data from all subjects, using self-reports of emotion as ground truth. Because the frequency of self-reported emotions was non-uniform, chance agreement between self-report and neural models was calculated based on the product of marginal frequencies, under the assumption of independent observer classifications [59]. Inference on the observed classification accuracy was tested against this value using the binomial distribution *B*(480, 0.2147). Due to infrequent self-reports of surprise, fear, and anger, accuracy on individual models was not computed.

Scores were initially assessed by averaging the 10 s preceding each rating. Subsequent analyses increasing the window length up to 20 s did not alter results. Because the scores for congruent (*p* = 0.0186, Lilliefors test against normal distribution) and incongruent (*p* = 0.0453) trials exhibited non-normal distributions, Wilcoxon signed rank tests were used to test each sample against zero mean rank. The correspondence between the frequencies of classification labels from self-report and neural decoding was assessed by computing the Pearson correlation for each subject. The correlation coefficients were Fisher transformed and tested against zero using a one-sample *t* test.

To ensure that population differences (i.e., inclusion of individuals with psychopathology) did not contribute to differences in the prevalence of emotions in the resting-state and experience sampling studies, we re-calculated the frequency of classifications using repeated random subsampling of healthy participants in the resting-state sample (1,000 iterations, sampling 21 participants without replacement). The average correlation between the healthy subsamples and the full sample was very high (r_avg_ = .981, s.d. = .013), making it unlikely that clinical status accounts for differences in the frequency of classifications across studies.

## Supporting Information

S1 Fig Importance maps for resting-state experiment. Parametric maps of t-statistics (one-sample t-test against 0) showing voxels whose activation (either positive or negative) contributed towards classification of (A) content, (B) amusement, (C) surprise, (D) fear, (E) anger, (F) sad, and (G) neutral states. Importance maps were created for each subject by taking voxel-wise product of classification weights and the mean activity of time-points assigned the corresponding label. Voxels are thresholded at an FDR corrected *q* = .05. The raw fMRI resting state data can be obtained from https://www.haririlab.com/projects.
(TIF)

S2 Fig Null frequency distributions for the classification of all seven emotional states (n = 499). (A) Colored distributions reflect the average frequency over 100 iterations of wavelet resampling. The mean, 25th and 75th percentiles are indicated by black lines. The solid gray line indicates the number of trials which would occur from random guessing. The range of the axes are matched to those in Fig 2B for ease of comparison. (B) Distribution of χ^2^ statistics across 100 iterations (Friedman test against uniform distribution), compared to that of the unpermuted data (solid red line). The data underlying this figure can be found in S1 Data. The raw fMRI resting state data can be obtained from https://www.haririlab.com/projects.
(TIF)

S3 Fig ℓ2-norms of models and data. (A) ℓ2-norms computed for each of the neural biomarkers of emotion. The ℓ2-norm is calculated by taking the square root of the sum of squared deviations across all voxels (i.e., Euclidean distance). These norms do not vary strongly across emotion models, indicating that the outputs of classifiers are typically on the same scale. (B) Mean (s.d.) of ℓ2-norms computed on the resting state data (n = 499 subjects). Each time-point reflects a single data acquisition lasting two seconds. Solid vertical line demarcates scans from the first and second run. The data underlying this figure can be found in S1 Data. The raw fMRI resting state data can be obtained from https://www.haririlab.com/projects.
(TIF)

S4 Fig Logistic regression models predicting the frequency of emotional states from individual difference measures. (A) Associations between self-reported anxiety and fear classifications (left) and between depressive symptoms and sad classifications (right). (B) Associations between the NEO scores for the Anxiety subfacet and fear classifications (left), between Angry Hostility and anger classifications (middle), and between Depression and sad classifications (right). Solid curves reflect the best fitting binomial model, semi-transparent curves reflect variation about the mean estimated by bootstrap resampling. Correlations between the estimated and observed classification frequencies are displayed on the upper right of each panel. The data underlying this figure can be found in S1 Data. The raw fMRI resting state data can be obtained from https://www.haririlab.com/projects.
(TIF)

S1 Table Friedman’s ANOVAs for changes in classification scores over time. (XLSX)

S1 Data Raw data underlying plots in Fig 1 (panel B), Fig 2 (panel B), Figs 3 and 4 (panels A and B), Fig 5 (panels B and C), S2 Fig (panels A and B), S3 Fig (panels A and B), and S4 Fig (panels A and B). (XLSX)

S1 Text Data sharing restrictions explanation. (DOCX)

## Abbreviations

CESD: Center for Epidemiologic Studies Depression Scale
NEO-PI-R: Revised NEO Personality Inventory
STAI-S: State-Trait Anxiety Inventory State Version
