# Supplementary figures and images for: Decoding Spontaneous Emotional States in the Human Brain
Source: PLoS Biol. 2016 Sep 14;14(9):e2000106. doi: 10.1371/journal.pbio.2000106 (PMC5023171; doi:10.1371/journal.pbio.2000106)

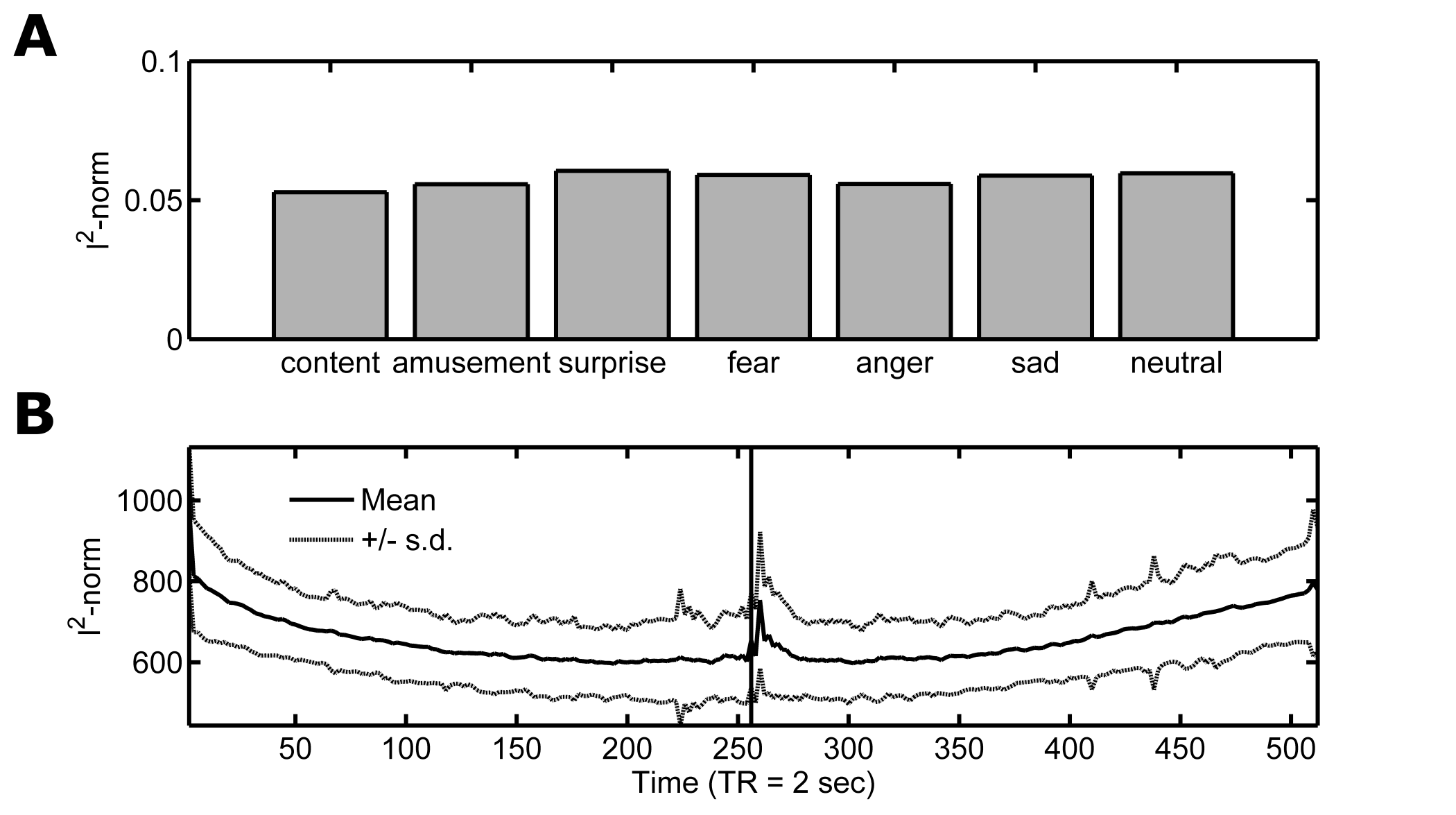

Supplement: S3 Fig — (A) ℓ2-norms computed for each of the neural biomarkers of emotion. The ℓ2-norm is calculated by taking the square root of the sum of squared deviations across all voxels (i.e., Euclidean distance). These norms do not vary strongly across emotion models, indicating that the outputs of classifiers are typically on the same scale. (B) Mean (s.d.) of ℓ2-norms computed on the resting state data (n = 499 subjects). Each time-point reflects a single data acquisition lasting two seconds. Solid vertical line demarcates scans from the first and second run. The data underlying this figure can be found in S1 Data. The raw fMRI resting state data can be obtained from https://www.haririlab.com/projects. (TIF) [file pbio.2000106.s003.tif]

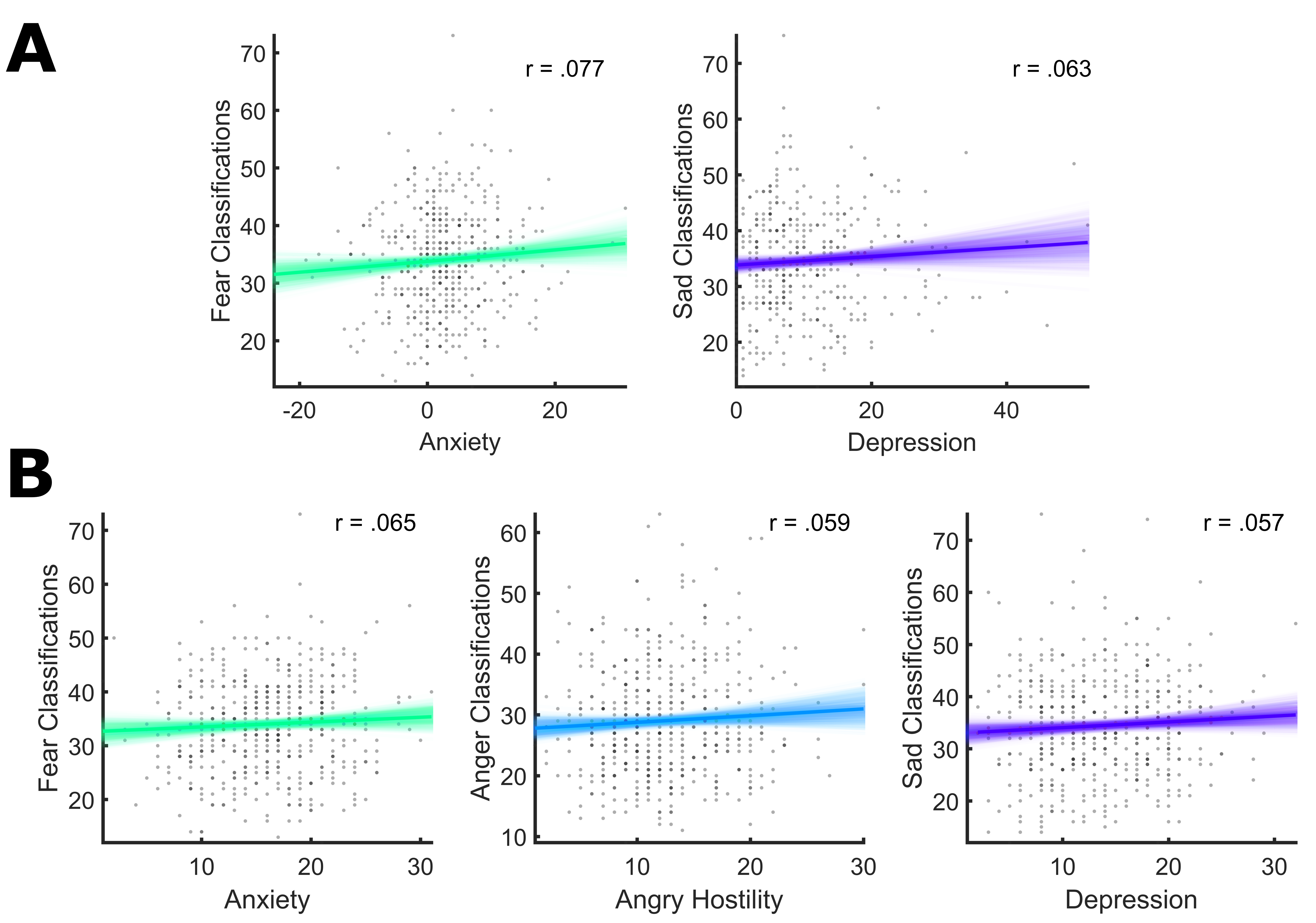

Supplement: S4 Fig — (A) Associations between self-reported anxiety and fear classifications (left) and between depressive symptoms and sad classifications (right). (B) Associations between the NEO scores for the Anxiety subfacet and fear classifications (left), between Angry Hostility and anger classifications (middle), and between Depression and sad classifications (right). Solid curves reflect the best fitting binomial model, semi-transparent curves reflect variation about the mean estimated by bootstrap resampling. Correlations between the estimated and observed classification frequencies are displayed on the upper right of each panel. The data underlying this figure can be found in S1 Data. The raw fMRI resting state data can be obtained from https://www.haririlab.com/projects. (TIF) [file pbio.2000106.s004.tif]
