# Supplementary material for: Decoding Spontaneous Emotional States in the Human Brain
Source: PLoS Biol. 2016 Sep 14;14(9):e2000106. doi: 10.1371/journal.pbio.2000106 (PMC5023171; doi:10.1371/journal.pbio.2000106)
Supplement: S1 Text — (DOCX) [file pbio.2000106.s007.docx]

**Introduction:**

We seek a careful balance between the benefits of data-sharing in research and any potential risks to study participants. Seeking this balance is aided by consultation of research and policy on data sharing. For example, in August 2014, NIH issued its Genomic Data Sharing (GDS) Policy. This policy is intended for investigators who intend to generate large-scale genomic and phenotypic data from federally funded new collections, and the Duke Neurogenetics Study (DNS) is not among that group. Nevertheless, we think the basic principles of the GDS Policy apply well to data sharing in general across many types of data. To explain our own policy, we draw on published materials from the GDS Policy (<http://osp.od.nih.gov/under-the-poliscope/2015/08/genomic-data-sharing-two-part-series>). This document is intended to give interested parties a full explanation of our data sharing policy at the current time, and the rationales behind it. The document ends by describing the requirements for accessing DNS data.

**The 2014 NIH GDS Policy** establishes that data-sharing can only occur with the advance consent of the participants, even if the datasets generated have been de-identified.  NIH now takes this approach to consent because formal research into participants’ preferences document that participants expect to be asked for permission before scientists use and share their de-identified data for research (for a special issue on this research see The End of Privacy, *Science* 22 Feb 2015, www.sciencemag.org). Moreover, as has been well-documented, the risk of re-identification of data is no longer a theoretical possibility and re-identified data could potentially be used to discriminate against or stigmatize participants, their families, or groups.  As such, it is no longer tenable for scientists to hold that anonymization is still achievable or to allow unrestricted sharing of “de-identified” datasets without consent on the premise that de-identified use is without risk to the donor.  The GDS Policy urges that the research enterprise must begin to respect the wishes of participants in relation to data access.

**We have not sought informed consent for unrestricted data sharing** because data from the DNS have been deemed by the Duke IRB as being in a **high-risk** category that precludes making the data set available for unrestricted unsupervised open-access data sharing. Consent documents for the study inform each study member that “Your data are held in strict confidence,” and “Only staff members of the Research Unit will have access to your data.” This means that the DNS participants have not at this point given their informed consent for unrestricted data sharing, and therefore data deriving from them cannot be made available for unrestricted use.

There are two main reasons for our approach to informed consent and data sharing. We note each below.

1. **Risk of mental pain and suffering.** The research team and IRBs recognize the risk to study members of mental pain and suffering from worry about the security of their data. The DNS data set contains sensitive information regarding topics concerning participants’ IQ, health behaviors, medical and psychiatric history. Unusual in research, the data set includes information divulged by study participants in confidential interviews about, for example, their lifetime history of mental disorders, suicidality, physical and sexual abuse victimisation, substance use, high-risk sexual behavior, life events such as abortions and divorce, and crimes committed. Additionally, our study includes sensitive information about participants’ families including their medical and psychiatric history. An ill-intentioned user could very easily misuse the data to illicitly identify individual study members and their families, and to expose confidential and potentially destructive details of their lives. The likelihood of any scientist doing this is immaterial. *What is material is the study members’ perceptions of the potential for data-security risk, and their concerns about it.*
2. **At-risk participants.** Substantial proportions of our sample belong to an at-risk group. More than half of our sample is children under the age of 21. It is standard IRB policy that such groups require a simple-to-understand, unconditional guarantee that all of their data are held in strict confidence by the research team. For these groups, trust is achieved by putting a face on who will use their data, and this is inconsistent with seeking consent for unrestricted data-sharing.

**The 2015 version of the Helsinki Declaration** addresses the potential for conflict between the aims of open-access data and the aims of human-subjects protection: Principle 8. While the primary purpose of medical research is to generate new knowledge, this goal can never take precedence over the rights and interests of individual research subjects (http://jama.jamanetwork.com/article.aspx?articleid=1760318).

**Our data-sharing policy** provides for investigators outside the study to access data used in this paper through collaboration.  We provide all such investigators with clean, well-documented data files and electronic data-set dictionaries. To ensure effective data sharing, the PI discusses detailed data-analysis plans with each investigator in advance and stays actively involved throughout each project.

**Access requirements.** Proposed data-analysis projects from qualified scientists require IRB approval at the applicants’ university, and provision for secure data access. We offer secure access on the Duke campus.

These requirements parallel those used by dbGap and the Health and Retirement Study. However, it is useful to know that unlike dbGaP and the HRS, the DNS has never been funded as a data provider.

**All scripts and analysis files for DNS published papers are available.**
